# Reduced emotion regulatory selection flexibility in post-traumatic stress disorder: converging performance-based evidence from two PTSD populations

**DOI:** 10.1017/S0033291721004670

**Published:** 2023-05

**Authors:** Naomi B. Fine, Noa Ben-Aharon, Daphna Bardin Armon, Zivya Seligman, Liat Helpman, Miki Bloch, Talma Hendler, Gal Sheppes

**Affiliations:** 1 Faculty of Social Sciences, School of Psychological Sciences, Tel-Aviv University, Tel-Aviv, Israel; 2 Sagol Brain Institute Tel-Aviv, Wohl Institute for Advanced Imaging, Tel-Aviv Sourasky Medical Center, Tel-Aviv, Israel; 3 Department of Psychiatry, Lotem Center for Treatment of Sexual Trauma, Tel Aviv Sourasky Medical Center, Tel Aviv, Israel; 4 Psychiatric Department, Tel Aviv Sourasky Medical Center, Tel-Aviv, Israel; 5 Department of Counseling and Human Development, University of Haifa, Haifa, Israel; 6 Sackler Faculty of Medicine, Tel-Aviv University, Tel-Aviv, Israel; 7 Sagol School of Neuroscience, Tel-Aviv University, Tel-Aviv, Israel

**Keywords:** PTSD, trauma, emotion regulation, selection, flexibility

## Abstract

**Background:**

Contemporary views of emotion dysregulation in post-traumatic stress disorder (PTSD) highlight reduced ability to flexibly select regulatory strategies according to differing situational demands. However, empirical evidence of reduced regulatory selection flexibility in PTSD is lacking. Multiple studies show that healthy individuals demonstrate regulatory selection flexibility manifested in selecting attentional disengagement regulatory strategies (e.g. distraction) in high-intensity emotional contexts and selecting engagement meaning change strategies (e.g. reappraisal) in low-intensity contexts. Accordingly, we hypothesized that PTSD populations will show reduced regulatory selection flexibility manifested in diminished increase in distraction (over reappraisal) preference as intensity increases from low to high intensity.

**Methods:**

Study 1 compared student participants with high (*N* = 22) post-traumatic symptoms (PTS, meeting the clinical cutoff for PTSD) and participants with low (*N* = 22) post-traumatic symptoms. Study 2 compared PTSD diagnosed women (*N* = 31) due to childhood sexual abuse and matched non-clinical women (*N* = 31). In both studies, participants completed a well-established regulatory selection flexibility performance-based paradigm that involves selecting between distraction and reappraisal to regulate negative emotional words of low and high intensity.

**Results:**

Beyond demonstrating adequate psychometric properties, Study 1 confirmed that relative to the low PTS group, the high PTS group presented reduced regulatory selection flexibility (*p* = 0.01, 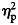 = 0.14). Study 2 critically extended findings of Study 1, in showing similar reduced regulatory selection flexibility in a diagnosed PTSD population, relative to a non-clinical population (*p* = 0.002, 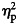 = 0.114).

**Conclusions:**

Two studies provide converging evidence for reduced emotion regulatory selection flexibility in two PTSD populations.

## Introduction

Post-traumatic stress disorder (PTSD) is a debilitating and tenacious condition, that involves a core impairment in the control or regulation of negative emotions (Karlsson & Sjöberg, 2009). Difficulties in emotion regulation are considered central to PTSD, because they predict the development and maintenance of the disorder, and are associated with more severe PTSD symptomatology (Boden et al., 2013; Forbes et al., 2020; Pencea et al., 2020).

The view on what constitutes emotion dysregulation in PTSD has shifted throughout the years. These shifts can be understood within a central engagement-disengagement regulatory classification that categorizes regulatory strategies as involving *Engagement* with emotional information processing and meaning-making *v. Disengagement* from emotional information processing and meaning avoidance (e.g. Parkinson & Totterdell, 1999; Roth & Cohen, 1986; Thayer & Lane, 2000).

Traditional views suggested that PTSD individuals over-utilize disengagement regulatory strategies such as avoidance at the expense of engagement regulatory strategies that promote emotional processing (Aldao, Nolen-Hoeksema, & Schweizer, 2010; Bonanno & Burton, 2013; Foa, Hembree, & Rothbaum, 2007; Foa & Kozak, 1986; John & Gross, 2004). However, later studies showed that in certain contexts, disengagement from stressful and traumatic events is associated with adaptive outcomes whereas engagement with emotional information processing is maladaptive (e.g. Bonanno, Keltner, Holen, & Horowitz, 1995; Chapman, Rosenthal, Dixon-Gordon, Turner, & Kuppens, 2017; Coifman, Bonanno, Ray, & Gross, 2007; See Park, 2010 for a review).

These mixed findings led to a new conceptual understanding that adaptive regulation of negative affective events may require the use of disengagement regulatory strategies in certain contexts and engagement regulatory strategies in other contexts (e.g. Aldao, Sheppes, & Gross, 2015; Bonanno & Burton, 2013; Bonanno, Papa, Lalande, Westphal, & Coifman, 2004 for review). This updated view suggests that individuals with PTSD may show reduced *regulatory selection flexibility* that manifests in reduced ability to choose engagement *v.* disengagement regulatory strategies in a manner that is sensitive to differing situational demands.

Despite the increasing conceptual agreement, empirical evidence for regulatory selection flexibility impairments in PTSD remains indirect. Specifically, one line of studies demonstrated that when PTSD individuals are instructed to *execute* engagement and disengagement strategies they show impaired execution flexibility (Bartholomew, Badura-Brack, Leak, Hearley & McDermott, 2017; Rodin et al., 2017). While important, these studies did not examine whether PTSD individuals fail to voluntarily *select* these strategies flexibly according to differing demands. A second line of studies showing that PTSD individuals self-report general impairments in the frequency of using regulatory strategies, did not assess active selection between strategies in different contexts (see Seligowski, Lee, Bardeen, & Orcutt, 2015 for a meta-analysis).

To fill these gaps the present two-study investigation was set to demonstrate deficits in regulatory selection flexibility in two different populations with PTSD symptomology. In doing so we concentrated on perhaps the most fundamental regulatory selection phenomenon, concerning the ability of individuals to flexibly select between regulatory disengagement and engagement strategies in a manner that is sensitive to differing emotional intensity levels.

Multiple studies have repeatedly demonstrated that healthy individuals show regulatory selection flexibility manifested in an increased preference to select distraction over reappraisal as intensity increases from low to high intensity (Sheppes, 2020 for a review). Specifically, in low-intensity contexts, healthy individuals strongly select to engage with emotional information and reinterpret its negative meaning via reappraisal, which is both effective in modulating mild emotional reactions, and more beneficial than a distraction for long-term adaptation (e.g. Thiruchselvam, Blechert, Sheppes, Rydstrom, & Gross, 2011). However, in high-intensity contexts, healthy individuals strongly select to disengage their attention via distraction, which effectively blocks potent emotional information and provides short term benefits (e.g. Shafir, Schwartz, Blechert, & Sheppes, 2015).

Relevant yet scarce support for the importance of flexible strategy selection in the context of trauma comes from a single study that found that exclusively among *non*-PTSD firefighters with impaired regulatory selection flexibility, higher traumatic exposure was associated with higher PTSD symptoms (Levy-Gigi et al., 2016).

In the current two-study investigation, Study 1 examined whether relative to college students with low post-traumatic symptoms, students with high post-traumatic symptoms that meet the clinical cutoff for PTSD would show reduced regulatory selection flexibility. Study 2 sought to critically extend the reduced regulatory selection flexibility findings of Study 1 in a clinical population of women with PTSD due to childhood sexual abuse (CSA). Emotion dysregulation is considered a strong predictor of CSA-PTSD (Ullman, Peter-Hagene, & Relyea, 2014), and it accounts for severe functional, and interpersonal impairments as well as to higher risk for ensuing psychopathology (Browne & Finkelhor, 1986; Cloitre, Miranda, Stovall-McClough, & Han, 2005; Kim & Cicchetti, 2010; Zlotnick et al., 1996). However, prior empirical evidence (e.g. Coffey, Leitenberg, Henning, Turner, & Bennett, 1996; Ehring & Quack, 2010; Griffing et al., 2006; Poole, Dobson, & Pusch, 2017) is restricted to impaired ability to *execute* different strategies, thus lacking crucial evidence regarding regulatory *selection* flexibility impairments for CSA-PTSD individuals.

To test our hypotheses, we validated a modified version of a classic performance-based regulatory selection paradigm (Sheppes, 2020 for review). In the modified paradigm participants are exposed to high and low negative intensity word stimuli and they behaviorally select whether they want to regulate their emotions using disengagement distraction or engagement reappraisal. Our use of high and low-intensity emotional words instead of pictorial stimuli previously used in the classic regulatory selection paradigm (c.f., Sheppes, Scheibe, Suri, & Gross, 2011), bypasses the requirement to use highly explicit and concrete traumatic content (e.g. mutilation pictures) in this vulnerable population (Kindt & Brosschot, 1997; Öhman & Soares, 1994; Wikström, Lundh, Westerlund, & Högman, 2004).

Hypotheses in both studies were identical. Compared to non-clinical individuals, individuals that meet the clinical cutoff for PTSD (Study 1) and women with PTSD due to CSA (Study 2) will show reduced regulatory strategy selection flexibility, manifested in a diminished increase in distraction (over reappraisal) preference as word stimuli intensity increases from low to high.

## Methods

Below we report how we determined our sample size, all data exclusions, all manipulations, and all measures that were collected in both studies. Study 1 was approved by the Institutional Review Board (IRB) and Study 2 was approved by the Medical Center Ethics (Helsinki) Committee.

### Study 1

#### Participants

As part of a standard departmental procedure, the first-year undergraduate student cohort (*n* = 317) signed informed consent and completed a battery of self-report measures at the beginning of the academic year, including the Post Traumatic Checklist (PCL-5, without criterion A) that assesses post-traumatic stress symptoms and constitutes our main group factor. In addition, students completed the Patient Health Questionnaire (PHQ-9) and the State-Trait-Anxiety Inventory (STAI) that assess depression and anxiety symptoms, respectively, in order to obtain common comorbidity measures. Students, unaware of the reason they were contacted, were invited to take part in the present study if their post-traumatic stress symptoms levels met a pre-defined clinical cutoff for PTSD (High PTS group: PCL-5 > 33, c.f., Rubin, Boals, & Berntsen, 2008; Weathers et al., 2013) or if they had minimal post-traumatic stress symptoms (Low PTS group: PCL-5 < 5). This relatively known ‘extreme group’ categorization design was chosen to maximize symptomatology differences (c.f., Azriel, Lazarov, Segal, & Bar-Haim, 2020; Shelby, Golden-Kreutz, & Andersen, 2008; Vail, Goncy, & Edmondson, 2019). From the large cohort, we identified 22 individuals that met the PCL clinical cut-off (high PTS group). To match the size of the high trauma group we chose 22 individuals with the lowest PCL scores (low PTS group). All participants had normal or corrected to normal vision, and were native Hebrew speakers, because understanding and implementing complex cognitive emotion regulation strategies require high verbal proficiency (c.f., Sheppes, 2014). For participation, students received academic credit or monetary compensation (~45 USD).

#### Procedure

Approximately 1–2 months following the mass testing, participants signed a written informed consent, completed the PCL-5 (including criterion A), followed by performing the modified performance-based regulatory selection paradigm. One week later participants completed the regulatory selection paradigm again in order to examine its test re-test reliability.^†^^1^

#### Clinical instruments

*Post-Traumatic Checklist* (PCL-5) – A 20 item self-administered inventory that indexes PTSD symptoms in the past month and is strongly recommended for the assessment of PTSD in undergraduate populations with mixed civilian trauma exposure (Adkins, Weathers, McDevitt-Murphy, & Daniels, 2008). Responses are rated on a scale of 0–4 and are summed to a total score. Cronbach's *α* in the current sample was *α* = 0.77.

*Patient Health Questionnaire* (PHQ-9) – A 9-item self-administered inventory indexing each of the DSM-IV depression criteria on a scale ranging from 0 to 3 (Kroenke, Spitzer, & Williams, 2001). Cronbach's *α* in the current sample was *α* = 0.85.

*State-trait Anxiety Inventory* (STAI) – A 20 item self-administered inventory of trait anxiety on a 4-point scale (Spielberger, 1983). Cronbach's *α* in the current sample was *α* = 0.78.

#### Modified performance-based regulatory selection word paradigm

*Stimuli*: 40 negative emotional words in Hebrew were selected from an Effective Norms for Hebrew Words database^2^ (Armony-Sivan, Cojocaru, & Babkoff, 2014). Low negative intensity words (*n* = 20, *M*_arousal_ = 4.8; s.d. = 0.61, *M*_valence_ = 2.5, s.d. = 0.6) differed significantly from high negative intensity words (*n* = 20, *M*_arousal_ = 7; s.d. = 0.48, *M*_valence_ = 1.9, s.d. = 0.47) in arousal, *t*(38) = −12.2, *p* < 0.001, and valence, *t*(38) = 3.67, *p* < 0.001. Low and high-intensity words were matched in word length, *t*(38) = 0.44, *p* > 0.66, and prevalence, *t*(38) = 0.58, *p* > 0.56. Emotional words included diverse negative content (e.g. ‘poverty’, ‘boredom’/ ‘death, ‘rape’, low/high intensity respectively) and were matched across the two intensity categories when possible. Previous studies with similar arousal and valence differences between low and high-intensity stimuli have demonstrated differential levels of emotional-response activation (e.g. Bradley, Codispoti, Cuthbert, & Lang, 2001; Shafir et al., 2015) and differential regulatory preferences (e.g. Sheppes et al., 2011; Sheppes, Brady, & Samson, 2014a, Sheppes et al. 2014b). Importantly, to provide further validation for our stimulus intensity categorization, at the onset of the study participants were presented with all words and rated their level of negative experience on a Likert scale (1 = not negative at all, 9 = extremely negative). As expected high-intensity words (*M* = 5.84 s.d. = 1.47) were rated as more negative than low-intensity words (*M* = 4.44 s.d. = 1.16), *F*(42) = 161.5, *p* < 0.001.

*Experimental paradigm:* Participants first learned how to implement disengagement-distraction and engagement-reappraisal (three examples for each instruction) and then practiced (six trials) choosing between them with an instruction to base their decision on the strategy which they assume would be more effective in reducing their negative emotional experience in response to each stimulus (c.f., Sheppes et al., 2011, 2014a, 2014b). Distraction instructions involved disengaging attention by producing unrelated neutral thoughts (i.e. visualizing daily activities or geometric shapes) (e.g. Shafir, Thiruchselvam, Suri, Gross, & Sheppes, 2016, 2017). Reappraisal instructions involved engaging with the processing of the emotional stimuli, but reinterpreting their negative meaning (i.e. thinking about less negative aspects of the situation or that the situation will improve over time) (Gross, 2014, 2002). In addition, participants were not allowed to form reality challenge reappraisals (i.e. interpret emotional events as unreal), since these reappraisals function as a form of disengagement (see Qi et al., 2017; Sheppes et al., 2014a, 2014b). Adherence to regulatory instructions during these phases was examined by asking participants to verbalize strategies out loud, during which corrective feedback was provided as needed.

**Fig. 1.**
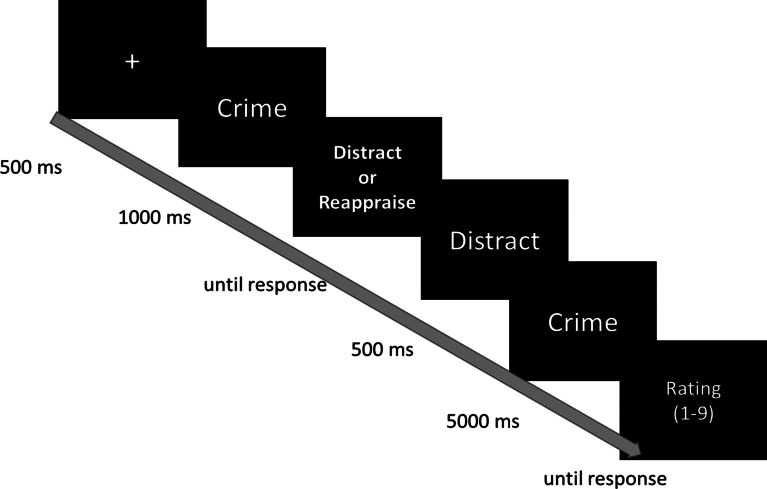
Illustration of a trial structure in the Modified Regulatory selection paradigm in which the participant saw a high emotional intensity word and selected disengagement distraction (ms = milliseconds).

The actual task consisted of 40 trials (divided into 2 equally long blocks, separated by a short break), during which words of low and high emotional intensity were presented in a random order, with the restriction that no more than two trials of the same emotional intensity category repeat in sequence. Each trial (see Fig. 1) began with a 500 ms fixation cross, followed by a 1000 ms preview of the emotional word. Then participants viewed a choice screen where they consciously indicated their regulatory selection between two fixed options- distraction or reappraisal by pressing a keyboard button that corresponded to each strategy (assignment of a button to the strategy was counterbalanced across trials), similarly to classic decision-making paradigms (e.g. Marewski & Schooler, 2011, for review). Following a reminder, cue preparing the participants to perform their chosen strategy (500 ms), the same word stimulus was presented again for 5000 ms, during which participants implemented their chosen strategy. The offset of each word was followed by a 1-to-9 Likert scale in which participants reported their level of negative emotional experience in response to the word (1 = ‘not negative at all’, 9 = ‘extremely negative’).^3^

#### Data analysis

To examine our main prediction^4^ regarding reduced regulatory selection flexibility among high relative to low PTS groups we employed a 2 × 2 mixed analysis of variance (ANOVA) with Group (High and Low PTS) as a between-subject factor, and Intensity (High and Low) as a within-participant factor, with the percentage of trials for which distraction was chosen (over reappraisal) as the dependent variable (Sheppes, 2020 for review). The expected two-way interaction was decomposed in a follow-up analysis that examined whether the high relative to low PTS group showed reduced regulatory selection flexibility manifested in a smaller increase in distraction (over reappraisal) preference from low to high intensity. For all analyses, we provide model fit estimates that include partial eta square and F-value.

## Results

### Demographics and reliability checks

Demographic and psychopathological characteristics by the group are presented in Table 1. Before addressing our main research question we wished to establish the internal and test-retest reliability indices of the modified regulatory selection word paradigm. First, meeting the standard acceptable value of the Kuder-Richardson 20 index (KR-20 = 0.5) in tasks with 20 or less binary items (Field, 2009; Dall'Oglio et al., 2010; Hinton, McMurray, & Brownlow, 2014), our low intensity [KR-20 = 0.71, 95% confidence interval (CI) 0.57–0.82] and high intensity (KR-20 = 0.53, 95% CI 0.31–0.71) showed adequate internal reliability. Second, participants' test-retest reliability indices showed a significant correlation across the weekly measurements for low intensity, *r*(42) = 0.38, *p* = 0.01, and high intensity, *r*(42) = 0.35, *p* < 0.001.

**Table 1.** Demographic and psychopathological characteristics by group

	High PTS (*n* = 22) Mean (s.d.)	Low PTS (*n* = 22) Mean (s.d.)	Statistics	
			*t/X^2^*	*p*
Demographics				
Age (s.d.)	22.9 (3.22)	24.31 (4.62)	*t* = 1.17	0.24
% Male	77%	72%	*X^2^* *=* *0.12*	0.78
Clinical characteristics				
PCL-5 (s.d.)	42.5 (8.05)	0.68 (1.17)	*t* = −24.08	0.000***
PHQ-9 (s.d.)	8.4 (6.56)	9.9 (5.82)	*t* = 0.8	0.42
STAI-T (s.d.)	50.72 (10.6)	35.45 (7.2)	*t* = −5.58	0.000***

PTS, Post Traumatic Symptoms; PCL-5, Post Traumatic Checklist; PHQ-9, Patient Health Questionnaire; STAI-T, State-Trait-Anxiety Inventory.

**p ⩽ 0.01, ***p ⩽ 0.001.

### Reduced regulatory selection flexibility in high relative to low PTS group

Replicating and extending prior regulatory selection findings obtained with images to word stimuli (see Sheppes, 2020 for a review), we found a significant main effect of intensity, indicating that participants' preference for distraction over reappraisal increased as the emotional intensity increased from low (*M* = 36.47%) to high (*M* = 56.7%) intensity, *F*(1,42) = 56.15, *p* < 0.0001, 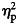 = 0.57.

**Fig. 2.**
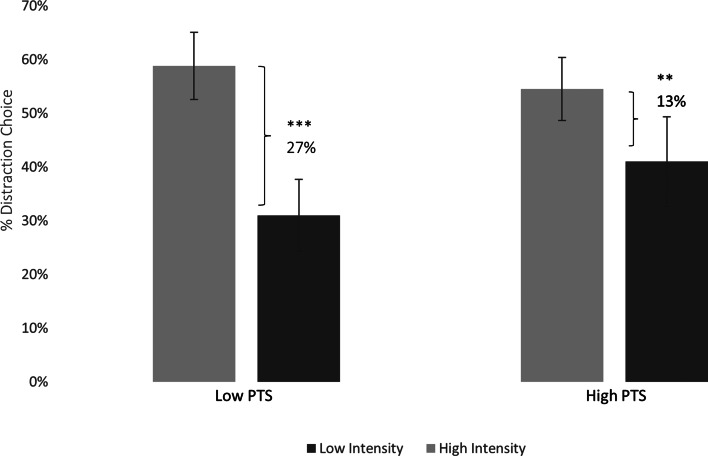
Performance-Based Emotion Regulatory Selection Flexibility in Low and High PTS groups. Percentage signifies Regulatory Selection Flexibility. Error bars represent 95% CIs. ** *p* ⩽ 0.01, ***p ⩽ 0.001.

Importantly, consistent with our main hypothesis, we found a significant two-way interaction between Group and Intensity, *F*(1,40) = 6.81, *p* = 0.01, 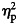 = 0.14, 95% CI 0.1–0.18 (See Fig. 2). Planned follow up analyses confirmed that the low PTS group demonstrated a robust *Regulatory Selection Flexibility* pattern, manifested in a 27% increase in distraction choice from low intensity (*M* = 31.59%, s.d. = 16.06%) to high intensity (*M* = 58.86%, s.d. = 15.88%), *F*(1,42) = −9.99, *p* < 0.000001. By contrast, the magnitude of the regulatory selection flexibility pattern in the high PTS group was less than half, and manifested in only a 13% increase in distraction choice from low intensity (*M* = 41.36%, s.d. = 2.03%) to high intensity (*M* = 54.54%, s.d. = 14.38%) *F*(1,42) = −7.79, *p* = 0.005.^5^

### Study 2

#### Participants

Given the supporting findings in Study 1 and given the similar design in both studies, for Study 2 we were able to determine the sample size with a formal a-priori power analysis. Using G*Power (Campbell & Thompson, 2012), applying the conventional power of 0.8, alpha of 0.05 and the observed effect size of the interaction from Study 1 (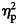 = 0.14), the analysis pointed to a required sample size of 27 participants in each group in order to detect a reliable effect.

Study 2 was conducted as part of a larger study,^6^ that included 31 female participants (*M*_age_ = 34.20, s.d. = 7.49; range = 22–50), with a history of recurrent CSA and an ascertained diagnosis of PTSD on the Clinician-Administered PTSD Scale (CAPS-5), but with no history of neurological disorder, psychosis, or current substance dependence. To match the clinical sample size, 31 non-clinical matched female controls (*M*_age_ = 31.51; s.d. = 6.43; range = 19–45) were recruited via electronic flyers, resulting in 62 participants for the final sample. Non-clinical participants completed the Life Events Checklist (LEC, self-report screening measure for trauma exposure) to ensure they did not meet criterion A and to specifically verify they were not exposed to sexual trauma (*M*_LEC # trauma_ = 0.16, s.d. = 0.11) and the Post Traumatic Checklist (PCL-5) in order to confirm they did not meet the clinical cutoff for PTSD (PCL-5 < 33; see Weathers et al., 2013). In addition, all participants completed several self-report measures including the Beck Depression Inventory (BDI-II) and State-trait Anxiety Inventory (STAI), that assess depression and anxiety symptoms, respectively, in order to obtain a general estimate of comorbidity. All participants had normal or corrected to normal vision and were native Hebrew speakers (c.f., Sheppes, 2014; Sheppes et al., 2011).

#### Procedure

Female PTSD-CSA participants that were in ongoing treatment in an out-patient clinic provided written informed consent according to the Medical Center Ethics Committee guidelines followed by participating in a CAPS assessment by a certified psychologist. Within a week after ascertaining DSM-5 PTSD diagnosis and completing self-report measures, in a separate session, the modified regulatory selection paradigm was administered. Similarly, non-clinical control participants provided written informed consent, completed self-report measures, and then completed the modified regulatory selection paradigm.

#### Clinical instruments

*Clinician-Administered PTSD Scale* (CAPS): We used the gold standard structured clinician interview for assessing PTSD diagnosis and symptom severity. We administered a version of the CAPS that combines DSM-IV and DSM-5 criteria in order to maintain continuity between classifications (Friedman, Kilpatrick, Schnurr, & Weathers, 2016; Hoge, Riviere, Wilk, Herrell, & Weathers, 2014, 2016). The CAPS contains explicit, behaviorally anchored probes for each of the 17 PTSD symptom criteria of the DSM-IV (on severity and frequency scale of 0–4), and 20 symptoms of the DSM-5 (on a severity scale of 0–4). Cronbach's *α* for the current sample was *α* = 0.73, *α* = 0.68 for CAPS-4 and-5 respectively.

*Post Traumatic Checklist* (PCL-5) and *State-trait Anxiety Inventory* (STAI) – See details in Study 1. Cronbach's *α* for the current sample was *α* = 0.937 and *α* = 0.81, respectively.

*Beck Depression Inventory* (BDI-II)–A 21 item self-administered inventory of depression symptoms and their respective intensity on a 4-point scale (Beck, Steer, & Carbin, 1988). Cronbach's *α* for the current sample was *α* = 0.86.

*The Life Events Checklist* (LEC) - The LEC is the self-report trauma assessment portion of the CAPS (Blake et al., 1995; Weathers, Keane, & Davidson, 2001) that assesses exposure to 16 traumatic events known to potentially result in PTSD. Items that were personally endorsed (‘the event happened to me’) receive a score of 1 and are summed to a total score.

#### Modified performance-based regulatory selection word paradigm

*Stimuli:* Word stimuli were identical to those used in Study 1. In order to provide further validation for stimuli categorization into low and high intensity, at the onset of the experimental procedure (identical to Study 1), participants were presented with all words and rated their level of negative experience on a Likert scale (1 = not negative at all, 9 = extremely negative). As expected, and replicating Study 1 findings, high-intensity words (*M* = 6.66, s.d. = 1.07) were rated as more negative than low-intensity words (*M* = 4.63, s.d. = 1.31), *F*(60) = 221.24, *p* < 0.0001.

*Experimental paradigm, and procedure:* Experimental paradigm and task procedure in Study 2 were identical to Study 1 except for the following changes. In the present study, in order to further verify regulatory choice adherence, 15% of the trials were randomly followed by a screen instructing participants to write a sentence describing how they implemented the strategy they chose (c.f., Sheppes et al., 2011). A judge who was blind to participants' choices (i.e. participants' button presses) coded the sentences as distraction or reappraisal. As expected and congruent with prior findings, levels of agreement approached a perfect score (96.9% accuracy), indicative of adequate adherence (e.g. Levy-Gigi et al., 2016; Sheppes et al., 2011).

#### Data analysis

Data analysis in study 2 was identical to Study 1.

## Results

### Demographics and reliability checks

Demographic and psychopathological characteristics by the group are presented in Table 2. Before addressing our main research question, as in Study 1, we calculated the internal reliability index of the modified regulatory selection word paradigm. Meeting the standard acceptable value of the Kuder-Richardson 20 index (KR-20 = 0.5) in tasks with 20 or less binary items (Field, 2009; Dall'Oglio et al., 2010; Hinton et al., 2014), our low-intensity KR-20 = 0.68 (95% CI 0.56–0.79) and high-intensity KR-20 = 0.75 (95% CI 0.65–0.83) showed adequate internal reliability.

**Table 2.** Demographic and psychopathological characteristics by group

	PTSD (*n* = 31) Mean (s.d.)	Non-Clinical (*n* = 31) Mean (s.d.)	Statistics	
			*T*	*p*
Demographics				
Age (s.d.)	34.20 (7.49)	31.51 (6.43)	1.48	0.14
Years of education (s.d.)	13.83 (2.64)	17.03 (3.13)	−4.29	0.000***
Clinical Characteristics				
PCL-5 (s.d.)	41.7 (17.89)	5.038 (10.36)	4.36	0.000***
CAPS-5 (s.d.)	76 (17.23)	-		
CAPS-4 (s.d.)	43.64 (8.91)	-		
BDI-II (s.d.)	23.73 (12.22)	5.89 (8.04)	6.43	0.000***
STAI (s.d.)	51.33 (13.73)	34.78 (10.78)	5.08	0.000***
LEC # trauma types		0.16 (0.11)		

PTSD, post-traumatic stress disorder group; CAPS, Clinician-Administered PTSD Scale; PCL-5, Post Traumatic Checklist; BDI-II, Beck Depression Inventory; STAI, State-Trait-Anxiety Inventory; LEC, # trauma types, Life Event Checklist.

**p ⩽ 0.01, *** p ⩽ 0.001.

### Reduced regulatory selection flexibility in PTSD relative to non-clinical group

Replicating prior and Study 1 findings, we found a significant main effect of intensity, indicating that participants' preference for distraction over reappraisal increased as the emotional intensity increased from low (*M* = 28.75%) to high (*M* = 51.56%) intensity, *F*(1,60) = 93.53, *p* < 0.001, 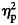 = 0.609.

**Fig. 3.**
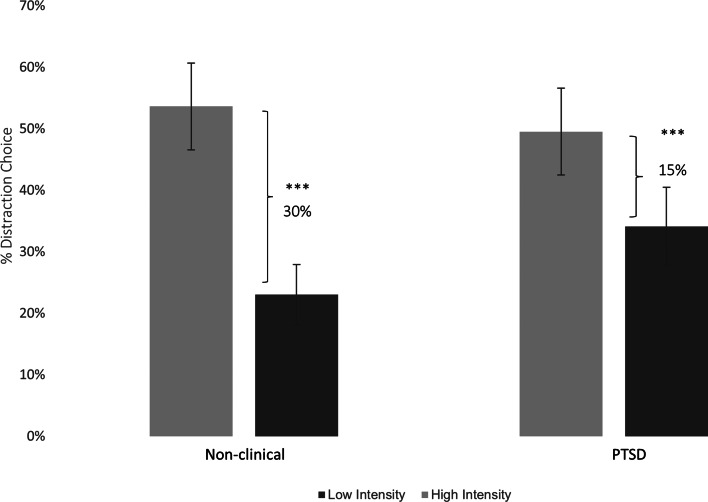
Performance-Based Emotion Regulatory Selection Flexibility in non-Clinical and PTSD groups. Percentage signifies Regulatory Selection Flexibility. Error bars represent 95% CIs. ** *p* ⩽ 0.01, *** p ⩽ 0.001.

Importantly, consistent with our main hypothesis, Study 2 extended Study 1 findings to a clinically diagnosed PTSD sample, and demonstrated converging evidence in showing a significant interaction between Group and Intensity, *F*(1,60) = 10.07, *p* = 0.002, 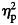 = 0.114, 95% CI −0.21 to 0.51 (See Fig. 3). Planned follow up analyses confirmed that the non-clinical group demonstrated a robust *Regulatory Selection Flexibility* pattern, manifested in a 30% increase in distraction choice from low intensity (*M* = 23.32%, s.d. = 14.13%) to high intensity (*M* = 53.61%, s.d. = 2.01%), *F*(1,60) = −9.12, *p* = 0.0001. By contrast, the magnitude of the regulatory selection flexibility pattern in the PTSD group was half in size, manifested by only a 15% increase in distraction choice from low intensity (*M* = 34.19%, s.d. = 2.18%) to high intensity (*M* = 49.51%, s.d. = 2.05%), *F*(1,60) = −4.52, *p* = 0.0002.

## General discussion

Despite a growing conceptual agreement that adaptive regulation involves flexibly matching emotion regulatory strategies to situational demands, empirical evidence of reduced *Regulatory Selection Flexibility* in PTSD is lacking. The present study demonstrated for the first-time reduced performance-based Regulatory Selection Flexibility in two different populations with PTSD symptoms. This impairment was manifested in reduced ability to flexibly choose engagement *v.* disengagement regulatory strategies in a manner that is sensitive to differing affective intensity demands. Specifically, Study 1 modified a performance-based regulatory selection paradigm using low and high-intensity affective word stimuli, and showed adequate internal reliability and significant test-retest reliability. Importantly, Study 1 confirmed hypotheses in showing that relative to college students with low PTS symptoms, students with high PTS symptoms presented reduced regulatory flexibility that was manifested in a smaller increase in distraction (over reappraisal) preference from low to high intensity. Extending Study 1 findings, Study 2 investigated a CSA-PTSD population that its hallmark deficit is emotional dysregulation. Mirroring findings from Study 1, Study 2 showed that relative to non-clinical women, women with a diagnosis of CSA-PTSD showed reduced regulatory flexibility that was demonstrated in a smaller increase in distraction (over reappraisal) preference from low to high intensity.

Taken together, findings from both studies provide important empirical support for the conceptual notion that PTSD individuals lack adaptive emotion regulation that requires the use of disengagement regulatory strategies in high-intensity contexts and engagement regulatory strategies in low-intensity contexts (e.g. Aldao et al., 2015; Bonanno & Burton, 2013; Bonanno et al., 2004 for review). Specifically, regulatory selection flexibility entails that in low-intensity contexts, individuals would predominantly select to engage with emotional information and reinterpret its negative meaning via reappraisal (e.g. Thiruchselvam et al., 2011), whereas in high-intensity contexts, individuals would predominantly select to disengage attention via distraction (Sheppes, 2020 for review).

Diverting from this healthy pattern, reduced regulatory selection flexibility in PTSD involves failing to maximize the benefits of selecting disengagement distraction to manage high-intensity events and or engagement reappraisal to cope with low-intensity events. Specifically, overly selecting disengagement regulation in low-intensity contexts precludes the long-term benefits of engaging with and making meaning of affective situations, and overly selecting engagement regulation in high-intensity contexts precludes the short-term benefits of warding off overwhelming negative emotions via disengagement strategies.

What might explain the reduced regulatory selection flexibility in PTSD? One possible explanation suggests that PTSD individuals lack available cognitive resources, which manifest in the decreased choice of strategies that are effortful to implement such as engagement reappraisal (c.f., Milyavsky et al., 2019; Sheppes et al., 2014a, 2014b). However, a general lack of resources can only partially explain the present findings. Specifically, the reduced regulatory selection flexibility in PTSD was indeed manifested in selecting less effortful reappraisal in low intensity, but PTSD individuals selected more reappraisal in high intensity. Therefore, it is possible that PTSD individuals may lack the cognitive resources required to alternate their regulatory selections between distraction and reappraisal to differing intensities of affective events.

The replicability and robustness of our findings, together with a firm theoretical background suggests that flexible regulatory selection may constitute an important underlying mechanism in PTSD. Accordingly, improving regulatory selection flexibility should be added to canonical clinical interventions that involve general efforts to improve regulatory selection (e.g. Berking, Ebert, Cuijpers, & Hofmann, 2013; Linehan, 2015).

Despite the replicable findings and novelty of the present investigation, it is important to mention several limitations and future directions. First, although groups categorization was based on well-defined parameters of PTS symptoms (Study 1) and PTSD diagnosis (Study 2), we cannot fully determine whether regulatory selection flexibility is a specific mechanism for PTSD or related to more general psychopathology. While additional analyses (see online Supplemental Materials) that covaried depressive and anxiety symptoms provided preliminary support for reduced regulatory selection flexibility that is specific to PTSD, future studies should further examine potential moderators that are associated with PTSD, such as comorbidities, gender, trauma type and trauma history.

Second, a possible limitation relates to the cross-sectional design of our study which does not allow to test whether selection flexibility impairment is an antecedent or consequence of PTSD symptoms (see Kring, 2008, for a review). Specifically, regulatory selection flexibility can be antecedent to PTSD and hence may predict PTSD symptoms. Alternatively, it can be a consequence such that individuals with PTSD symptoms may become less emotionally flexible.

Third, the current study has some psychometric limitations. In self-report measures, we found relatively low Cronbach's alphas for PCL in Study 1 and CAPS-5 in Study 2. Nevertheless, obtaining similar regulatory selection findings in Study 2 where internal consistency of the PCL was high (*α* = 0.937) strengthens our confidence in the results of Study 1. In the performance-based Regulatory Selection paradigm, we found relatively low (albeit significant) test-retest correlations. Accordingly, conclusions regarding reduced regulatory selection flexibility in PTSD should be limited to the group level rather than the individual level (c.f., Berger, 2006).

Fourth, while the present findings demonstrate reduced regulatory selection flexibility in PTSD, the affective consequences of this selection deficit cannot be accurately evaluated using our paradigm (see Footnote 3). Accordingly, future studies should consider combining the regulatory selection paradigm with a regulatory implementation paradigm (where participants are instructed which strategy to employ on each trial) that accurately assesses affective consequences. Of possible affective consequence measures, future studies should consider electrophysiological measures of regulatory success (e.g. late positive potentials) that have been proven to adequately reveal the consequences of different strategies across varying intensities (e.g. Shafir et al., 2015).

Lastly, although the present study investigated the two most established regulatory engagement and disengagement strategies and the central emotional intensity situational factor (Sheppes, 2020), future studies may consider testing other strategies along the engagement disengagement continuum as well and other cognitive and motivational factors to further establish regulatory selection flexibility impairments in PTSD.

## Footnotes

† The notes appear after the main text.

## Notes

1 In addition to completing the modified regulatory selection word paradigm in each session, participants also completed a modified regulatory selection pictorial paradigm for pilot purposes. Due to ethical considerations, the pictorial stimuli included images that are less graphic and lower in intensity, compared to stimuli commonly used in prior studies with healthy subjects (e.g. Levy-Gigi et al., 2016; Sheppes, 2014; Sheppes, Suri, & Gross, 2015). The pictorial task was not administered in Study 2.

2 All words are included in online Supplemental Materials (see online Supplementary Table S1).

3 It has been widely documented (Scheibe, Sheppes, & Staudinger, 2015; Sheppes, 2020 for review) that the regulatory selection paradigm is not designed, and thus cannot provide, accurate assessment of the affective consequences of regulatory selection. This is because inferences about differential efficacy of regulatory strategies following regulatory selection (e.g. whether in a high intensity condition distraction regulatory decisions result in lower negative affect relative to reappraisal regulatory decisions) require equating stimuli's pre-choice emotional intensity for each of the two conditions (e.g. equating the initial negativity of stimuli that led to distraction relative to reappraisal regulatory decisions). In the regulatory selection paradigm experimental matching of intensity is not possible because participants freely select between strategies for each stimulus. Accordingly, since post-choice ratings are un-interpretable we made an a-priori decision to not analyze them. Note that post choice ratings are typically included in the paradigm in order to remind participants the goal of regulatory selection, which is to choose on each trial the regulatory option they think will assist them the most to reduce their negative emotional experience.

4 In both studies before performing data analyses, normal quantile-quantile (QQ) plots (Osborne & Overbay, 2004) verified that the assumption of normality was not violated and no outliers were detected (c.f., outlier detection approach Miller, 1991). Therefore, ANOVAs were used with all data maintained for analyses.

5 The supplemental materials include a clear statistical rationale and additional analyses that covary for depression and anxiety in Study 1 and Study 2, all supporting the results reported in the main text.

6 Participants in the PTSD group were enrolled to a larger randomized control trial investigating the effectiveness of a new treatment (see full procedure and protocol: ClinicalTrials.gov ID; NCT 04303533). The administration of the regulatory selection paradigm was conducted at baseline before participants received treatment. Participants received monetary compensation for their participation.

## Supplementary material

For supplementary material accompanying this paper visit https://doi.org/10.1017/S0033291721004670. click here to view supplementary material
