# Supplementary material for: Reduced emotion regulatory selection flexibility in post-traumatic stress disorder: converging performance-based evidence from two PTSD populations
Source: Psychol Med. 2021 Nov 29;53(7):2758–67. doi: 10.1017/S0033291721004670 (PMC10244008; doi:10.1017/S0033291721004670)
Supplement: Supplementary file 1 [file S0033291721004670sup001.docx]

**Supplementary Material**

Table S1. List of words. Note that words were presented in Hebrew and were equal in length. Due to differences between languages, translation to English occasionally resulted in slightly different and longer words.

| Low Intensity | High Intensity |
| --- | --- |
| Guilt | Aggression |
| Exhausting | Anxiety |
| Rubbish | Burglary |
| Diarrhea | Crime |
| Prison | Death |
| Nuisance | Distress |
| Poverty | Distruction |
| dependency | Enemy |
| Disorder | Eradication |
| Spit | Fear |
| Weakness | Horror |
| Infection | Loss |
| Filth | Dying |
| Absence | Murderer |
| Jealousy | Pain |
| Overweight | Rage |
| Boredom | Rape |
| Branding | Stabbing |
| Suspision | Trample |
| Sweat | Violence |

**Supplementary Analyses:** Despite its wide usage, it has been argued that analysis of covariance (ANCOVA) may be misused when trying to account for systematic (non-random) group differences (c.f., "Misunderstanding Analysis of Covariance", Miller, 2001). Specifically, because in the present studies random assignment of participants to groups cannot be made, differential anxiety and depression levels between groups likely reflect systematic (non-random) differences. Nevertheless and congruent with the importance of demonstrating PTSD symptom specificity, in Study 1 there were no significant differences in depression scores between groups. Accordingly, we were able to adequately employ a repeated measures ANCOVA with depression scores as a covariate that yielded the expected significant interaction between Group and Intensity, *F*(1,42)=6.25, *p*=0.017, $\text{η}$_p_^2^=0.13. This finding suggests that the observed regulatory selection flexibility differences found between groups go beyond depression scores and provide further evidence for PTSD disorder specificity.

Second, despite the possible misapplication of ANCOVAs in cases where covariates are uses to account for significant differences between groups, employing these analyses led to similar findings. Specifically, in both studies employing a repeated measures ANCOVAs while covarying anxiety and depression scores led to the expected significant interaction between Group and Intensity, Study 1: *F*(1,42)=7.02, *p*=0.011, $\text{η}$_p_^2^=0.14; Study 2: *F*(1,60)=4.428, *p*=0.04, $\text{η}$_p_^2^=0.071.

**References**

Miller, G. A. Miller, G. A. & Chapman, J. P. (2001). Misunderstanding analysis of covariance. *Journal of abnormal psychology*, *110*(1), 40. https://doi.org/10.1037/0021-843X.110.1.40
